# Generation of Pediatric Leukemia Xenograft Models in NSG-B2m Mice: Comparison with NOD/SCID Mice

**DOI:** 10.3389/fonc.2016.00162

**Published:** 2016-06-27

**Authors:** Anilkumar Gopalakrishnapillai, E. Anders Kolb, Priyanka Dhanan, Aruna Sri Bojja, Robert W. Mason, Diana Corao, Sonali P. Barwe

**Affiliations:** ^1^Nemours Center for Childhood Cancer Research, A.I. DuPont Hospital for Children, Wilmington, DE, USA

**Keywords:** patient-derived xenografts, NSG-B2m mice, pediatric leukemia, engraftment, survival

## Abstract

Generation of orthotopic xenograft mouse models of leukemia is important to understand the mechanisms of leukemogenesis, cancer progression, its cross talk with the bone marrow microenvironment, and for preclinical evaluation of drugs. In these models, following intravenous injection, leukemic cells home to the bone marrow and proliferate there before infiltrating other organs, such as spleen, liver, and the central nervous system. Moreover, such models have been shown to accurately recapitulate the human disease and correlate with patient response to therapy and prognosis. Thus, various immune-deficient mice strains have been used with or without recipient preconditioning to increase engraftment efficiency. Mice homozygous for the severe combined immune deficiency (*SCID*) mutation and with non-obese diabetic background (NOD/SCID) have been used in the majority of leukemia xenograft studies. Later, NOD/SCID mice deficient for interleukin 2 receptor gamma chain (*IL2Rγ*) gene called NSG mice became the model of choice for leukemia xenografts. However, engraftment of leukemia cells without irradiation preconditioning still remained a challenge. In this study, we used NSG mice with null alleles for major histocompatibility complex class I beta2-microglobulin (*β2m*) called NSG-B2m. This is a first report describing the 100% engraftment efficiency of pediatric leukemia cell lines and primary samples in NSG-B2m mice in the absence of host preconditioning by sublethal irradiation. We also show direct comparison of the engraftment efficiency and growth rate of pediatric acute leukemia cells in NSG-B2m and NOD/SCID mice, which showed 80–90% engraftment efficiency. Secondary and tertiary xenografts in NSG-B2m mice generated by injection of cells isolated from the spleens of leukemia-bearing mice also behaved similar to the primary patient sample. We have successfully engrafted 25 acute lymphoblastic leukemia (ALL) and 5 acute myeloid leukemia (AML) patient samples with distinct cytogenetic characteristics in NSG-B2m mice, with the purpose of generating pediatric ALL and AML xenografts for preclinical evaluation of drugs. Thus, our data support the use of NSG-B2m mouse model for leukemia engraftment and *in vivo* preclinical drug efficacy studies.

## Introduction

Acute leukemia is the most common malignancy in children. Leukemia is characterized by the proliferation of immature blasts in the bone marrow that gradually infiltrate the spleen, liver, lymph nodes, and sometimes the central nervous system (CNS). With ~3000 pediatric cases diagnosed in the US annually (1), acute lymphoblastic leukemia (ALL) originating from neoplastic lymphoid progenitors is more prevalent than acute myeloid leukemia (AML). ALL is classified as B-cell ALL (B-ALL) or T-cell ALL (T-ALL) depending on the specific immunophenotypic characteristic of the progenitor clone, B-cell precursor or T-cell lineage, respectively. AML arises from genetic changes in myeloid progenitor cells leading to their aberrant expansion. The incidence rate of AML is ~7 per 1 million children per year (2).

Primary leukemia cells cannot be cultured *in vitro* for long periods, but leukemia xenografts have proven extremely useful, not only for passaging primary samples but also for the modeling of the human disease in mice (3). In these models, human cell lines or leukemia cells isolated from patients are intravenously injected into immunodeficient mice to generate systemic disease. Leukemia cells engraft and proliferate in the bone marrow followed by infiltration into the spleen, liver, and other organs including CNS (4). The progression of the disease in mice can be tracked in real time by sampling murine peripheral blood (5, 6). These models accurately recapitulate the disease characteristics, such as blast morphology, immunophenotype, and sites of organ infiltration (5). Hind limb paralysis is a common symptom owing to the infiltration of leukemic cells into the CNS in some mice (7), consistent with the involvement of the CNS in a small patient population (8, 9).

The suitability of xenograft models for preclinical testing of novel drugs or novel combinations of existing drugs was established by studies showing the correlation of xenograft drug responses with patient clinical outcome (6). Although the lack of a native immune system in these immune-deficient mouse hosts prevents the study of interaction between the tumor and the immune system, these mouse models can be effectively used for deciphering the role of the bone marrow microenvironment on leukemia cell growth and chemoresistance (10, 11). That leukemic cells alter the bone marrow niche to their liking and thereby disrupt normal hematopoiesis was demonstrated using these mouse models (12). The identification of a therapy-induced niche that supports the survival of cancer-propagating cells that ultimately lead to disease relapse was possible by using xenotransplantation of ALL cells in immune-deficient mice (13). Thus, the benefits of using leukemia xenograft models for understanding leukemia disease biology have been established (14).

Non-obese diabetic/severe combined immunodeficient (NOD/SCID) mice pre-conditioned with sublethal irradiation are the most commonly used recipients for the engraftment of patient-derived leukemic cells for preclinical testing (15). However, the engraftment efficiency is reported to be lower in the absence of irradiation pretreatment. This is believed to be due to the presence of innate immunity and remnants of the immune system in NOD/SCID mice. Some young adult mice can generate a few clones of B-cells and T-cells due to leakiness of the SCID mutation, although it is minimal in mice with the NOD background (16). To overcome this hurdle, other groups used NOD/SCID mice null for the major histocompatibility complex (MHC) class I molecule beta2-microglobulin gene (NS-β2m) (17, 18) or NOD/SCID mice with interleukin 2 receptor gamma gene (IL2Rγ) deletion (NSG) (19–21). We utilized NOD/SCID mice with deletions in both these genes (NSG-B2m) for establishment of xenograft mouse models. Although NSG-B2m mice have been used earlier for graft-versus-host disease studies (22, 23), ours was the first group to use this mouse model for generation of leukemia xenografts. Our data show that NSG-B2m mice support engraftment of primary human ALL and AML samples with diverse cytogenetic characteristics (Table 1) in the absence of irradiation preconditioning and at 100% engraftment efficiency.

**Table 1:** **Cytogenetic characteristics of patient samples engrafted**.

ID No.	Ethnicity	Age	Gender	Diagnosis	FISH	Karyotype
NTPL-20	African American	6	M	B-ALL	BCR/ABL1 translocation, p16 gene deletion	46XY, der(9)del(9)(p21.3)inv(9)(q32p21.2)ins(9;22)(q34;q11.2),der(22)t(9;22)(q34;q11.2)[13]/46XY[7]
NTPL-24	Caucasian	4	M	T-ALL	Negative	46XY, del(6)(q21),del(9)(p22)[cp7]/46XY[13]
NTPL-26	Caucasian	4	F	B-ALL	TEL/AML1 (ETV6/RUNX1) fusion, MLL gene deletion	47XX,+21c[cp12]/47,idem,del(9)(p21),del(11)(q23),−13,+mar[cp8]
NTPL 59	Caucasian	1	F	T-ALL	Negative	46XX
NTPL-60	African American	4	M	AML	AML1 and ETO amplification	46XY, der (14;21) (q10;q10) ?c, +21c [cp12]/48, idem, +8, +der (14;21) (q10; q10) [cp8]
NTPL-83	Caucasian	4	F	B-ALL	TEL/AML1 (ETV6/RUNX1) fusion	47XX, +21 [6]/46XX [14]
NTPL-84	Asian	8	F	B-ALL	Hyperdiploidy, p16 gene deletion	52XX,+X,+4,dic(4;6)(p12q11.2),+6,+14,+17,+21,+21[cp7]/46XX[13]
NTPL-87	Caucasian	14	M	B-ALL	Normal	46XY
NTPL-90	Not known	3	F	B-ALL	ETV6/RUNX1 fusion	46XX
NTPL-92	African American and Caucasian	5	F	B-ALL	E2A/PBX1 gene fusion	46XX, der(19)t(1;19)(q23;p13)[2]/46XX[18]
NTPL-103	Caucasian	4	F	B-ALL	Trisomy 21	47XX, +21
NTPL-104	Caucasian	11	F	AML	C/EBPalpha mutation	46XX
NTPL-109	Hispanic	3	M	B-ALL	E2A gene deletion	46XY
NTPL-119	Hispanic	3	F	B-ALL	RUNX1 amplification	46XX, ?ins(7;15) (?p15;?q12q26), −9, +mar[cp3]/46, XX [17]
NTPL-127	Caucasian	7	M	B-ALL	RUNX1 amplification	46XY
NTPL 137	Caucasian	7	F	B-ALL	Hyperdiploidy, trisomy 4, 10, tetrasomy 21	Pending
NTPL-138	Hispanic	4	M	B-ALL	Hyperdiploidy, p16 gene deletion	58XY, +X, +4, +6, +7, +10, +14, del(16)(q22), +17, +18, +21, +21 +2mar[cp6]/46, XY [14]
NTPL-150	Hispanic	7	M	B-ALL	Hyperdiploidy, p16 gene deletion, RUNX1 gain	51–52XY, +add(X)(22.3), i(8)(q10), dic(9;20)(p11.2;q11.2), del(9)(p21), +14, +18, +21, +21, +21, +21 [cp5/46, XY[15]]
NTPL-155	Hispanic	16	M	B-ALL	p16 gene deletion	46XY
NTPL-164	African American	4	M	B-ALL	p16 gene deletion	46XY, del(9)(p21)[cp2]/46, XY [18]
NTPL-168	African American	5	M	B-ALL	Negative	46XY
NTPL-216	Not known	2	F	B-ALL	ETV6/RUNX1 fusion, p16 gene deletion	46XX
NTPL 301	Caucasian	13	F	AML	Monosomy 5, monosomy 7, TEL deletion	42 ~ 43, X, t(2;16)(q21;p13.1),add(4)(q21),der(5)t(5;12)(q13;q11.2),−7,add(12)(p11.2),add(15)(q22,−17,−19,add(20)(p13),+1 ~ 2mar[cp8/42,sl,−13[cp5]]/42,sdl1,+del(13)(q12q14),−add(20)[2]/42,sdl2,−der(5),+add(7)(q22), ins(10)9p11.2)[2]/42,sl2,−7,der(13)t(7;13)(q11.2;p11.2)[2]/45,X,−X[1]
NTPL-313	Caucasian	6	F	B-ALL	ETV6/RUNX1 fusion	45X,−X,del(6)(q13q21),del(12)(p11.2),del(13)(q31),−18,+mar[cp6]/46,XX[14]
NTPL 315	Caucasian	14	M	T-ALL	Negative	46XY
NTPL-344	Hispanic	3	M	B-ALL	ETV6/RUNX1 fusion, ETV6 deletion	45XY,?dic(12;18)(p11.2;p11.2)[11]/46.xy[9]
NTPL-367	Hispanic	15	M	B-ALL	Negative	46XY
NTPL-386	Non-hispanic	2	M	AML	RUNX1 amplification	47XY,del(13)(q12q14),+21c[12]/47,ldem,l(7)(q10)[3]/47,XY,+21c[5]
NTPL-454	Caucasian	16	M	T-ALL	Negative	46XY
NTPL-511	Unknown	14	M	AML	Negative	47XY,+8[1]/46,XY[29]

## Materials and Methods

### Cell Lines and Patient Samples

AML-193 (CRL-9589), HL-60 (CCL-240), MV4;11 (CRL-9591), REH (CRL-8286), RS4;11 (CRL-1873) cells were obtained from American Type Culture Collection (ATCC), Manassas, VA, USA. Nalm6 cells were purchased from DSMZ-German Collection of Microorganisms and Cell Cultures, Braunschweig, Germany. RS4;11, REH, and Nalm6 cells were cultured in RPMI culture medium supplemented with 10% fetal bovine serum (FBS), 2 mM/L l-glutamine, 25 U/ml penicillin, and 25 μg/ml streptomycin. MV4;11 and HL-60 cells were cultured in IMDM culture medium with supplements listed above, except that 20% serum was used for HL-60 cells. AML-193 cells were cultured in IMDM with 5% FBS, 0.5 ng/ml insulin, 5 ng/ml transferrin receptor, and 5 ng/ml GM-CSF.

Primary leukemia samples collected under a Nemours Delaware Institutional Review Board (IRB) protocol approved by the Nemours Office of Human Subjects Protection were provided by the Nemours BioBank. Bone marrow aspirates or peripheral blood of patients treated at Nemours/Alfred I. duPont Hospital for Children were subjected to Ficoll (Ficoll Paque Plus, GE Healthcare Bio-Sciences, Pittsburgh, PA, USA) density gradient centrifugation. Cells isolated from the interphase had at least 90% of leukemic blasts as determined by flow cytometry.

### Antibodies

FITC-conjugated human CD45, CD10, CD19, CD3, CD33 and APC-conjugated mouse CD45 and human CD45 antibodies were obtained from Affymetrix eBioscience (San Diego, CA, USA).

### Flow Cytometry

Mouse peripheral blood was collected periodically by submandibular bleeding. Blood was stained with FITC-conjugated human CD45 and APC-conjugated mouse CD45 antibodies. The red blood cells (RBC) were lysed by using a multispecies RBC lysis buffer (Affymetrix eBioscience) containing ammonium chloride. Following RBC lysis, the number of cells stained specifically for either antibody (CD45+ cells) within a cell population gated for lymphocytes or myeloid cells (based on their forward and side scatter parameters) was determined by flow cytometry using a BD Accuri C6 flow cytometer, BD Biosciences, San Jose, CA, USA. The percentage of human cells in mouse peripheral blood was calculated using this formula – (No. of human CD45+ cells)/(No. of human CD45+ cells + No. of mouse CD45+ cells) × 100. The increase in this percentage over time was plotted and fitted to a non-linear regression curve. The time at which the percentage of human cells in mouse peripheral blood reached 1% was interpolated from this curve using Prism 6, GraphPad Software, San Diego, CA, USA.

For immunophenotypic characterization, blood sample from a single mouse (100–150 μl) was split (30–50 μl per tube). APC-conjugated human CD45 antibody was used in combination with FITC-tagged human CD10, CD19, and CD3 antibody for double staining. For cytoplasmic CD3 staining, blood was incubated with equal volumes of 1% paraformaldehyde for 15 min in the dark. Cells were permeabilized with 0.5% saponin for 15 min and stained with FITC-conjugated anti-CD3 antibody for 5 min. At this point, the RBC were lysed. The stained cells within the lymphocyte gate were analyzed by flow cytometry as described above.

### Xenograft Studies

Non-obese diabetic/severe combined immunodeficient and NSG-B2m mice were obtained from Jackson Laboratories, Bar Harbor, ME, USA. Leukemia cells (3–10 × 10^6^) were transplanted into NOD/SCID or NSG-B2m mice *via* tail-vein injections. Mice were maintained in the Nemours Life Science Center following the guidelines established by the Nemours Institutional Animal Care and Use Committee (IACUC). Disease progression was monitored by flow cytometry of mouse peripheral blood drawn periodically by submandibular bleeds. Mice were sacrificed by carbon dioxide asphyxiation using a method consistent with the euthanasia guidelines of the American Veterinary Medical Association, when they exhibited disease symptoms, such as increased leukemic burden, persistent weight loss, or hind limb paralysis. Following sacrifice, leukemic cells were harvested from the bone marrow and spleen, and enriched by Ficoll gradient centrifugation using Ficoll Paque Plus. All studies involving mice were approved by the Nemours IACUC.

### Statistical Analysis

GraphPad Prism software was used for comparison between two or more growth curves using the Extra sum-of-squares *F* test. Kaplan–Meir survival curves were compared by Log-rank (Mantel–Cox) test. A *p* value of less than 0.01 was considered statistically significant.

## Results

### ALL Cells Engraft Faster and at a Higher Efficiency in NSG-B2m Cells Compared to NOD/SCID Mice

**Figure 1:**
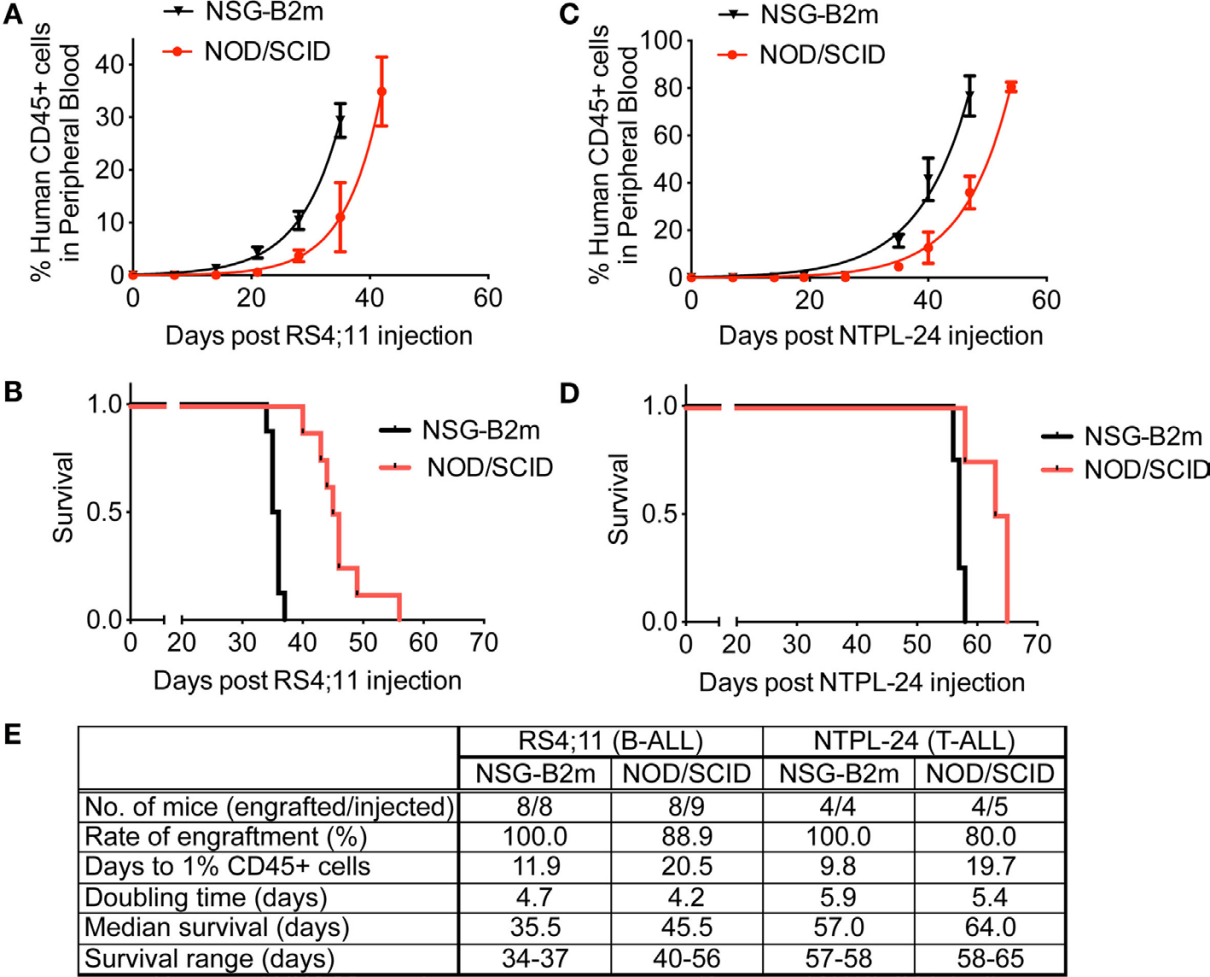
**NSG-B2m mice engraft faster and at a higher efficiency compared to NOD/SCID mice**. Growth curves showing the increase in the percentage of human CD45+ cells in mouse peripheral blood in mice injected with RS4;11 **(A)** or NTPL-24 **(C)** cells. Kaplan–Meier survival plots showing the survival of NSG-B2m or NOD/SCID mice following transplantation with RS4;11 **(B)** or NTPL-24 **(D)** cells. **(E)** Table summarizing the growth and survival curves described above.

We tested the engraftment rate and efficiency of an ALL cell line and a primary sample in NSG-B2m and NOD/SCID mice without the use of sublethal irradiation. Following intravenous injection of 3 × 10^6^ RS4;11 cells, the mice were monitored for disease progression by periodic bleeds. The percentage of human leukemic cells in mouse peripheral blood was determined by flow cytometry using species-specific antibodies. We observed that RS4;11 cells engrafted significantly faster in NSG-B2m mice as compared to NOD/SCID mice (Figure 1A, *p* < 0.0001). The estimated time taken to attain an average of 1% human CD45+ cells in mouse peripheral blood was 11.9 days in NSG-B2m mice compared to 20.5 days in NOD/SCID mice (Figure 1E). The engraftment efficiency was 100% in NSG-B2m mice, whereas it was 88.9% in NOD/SCID mice. Corresponding to the enhanced rate of engraftment, the RS4;11-injected NSG-B2m mice succumbed to disease significantly sooner than NOD/SCID mice (Figure 1B, *p* < 0.0001).

We also compared the engraftment rate and survival of NOD/SCID and NSG-B2m mice injected with 10 × 10^6^ cells of a primary T-ALL sample, NTPL-24. NSG-B2m mice engrafted significantly faster (Figure 1C, *p* < 0.0001), and their median survival was significantly lower (Figure 1D, *p* < 0.01). The rate of engraftment of NTPL-24 in NOD/SCID mice was also lower than in NSG-B2m mice (Figure 1E). Interestingly, the doubling time of both samples was similar in NSG-B2m and NOD/SCID mice suggesting that the initial lag in the engraftment precipitated into delayed engraftment. Taken together, these data indicate that NSG-B2m mice represent a better model for leukemia cell engraftment compared to NOD/SCID mice.

**Figure 2:**
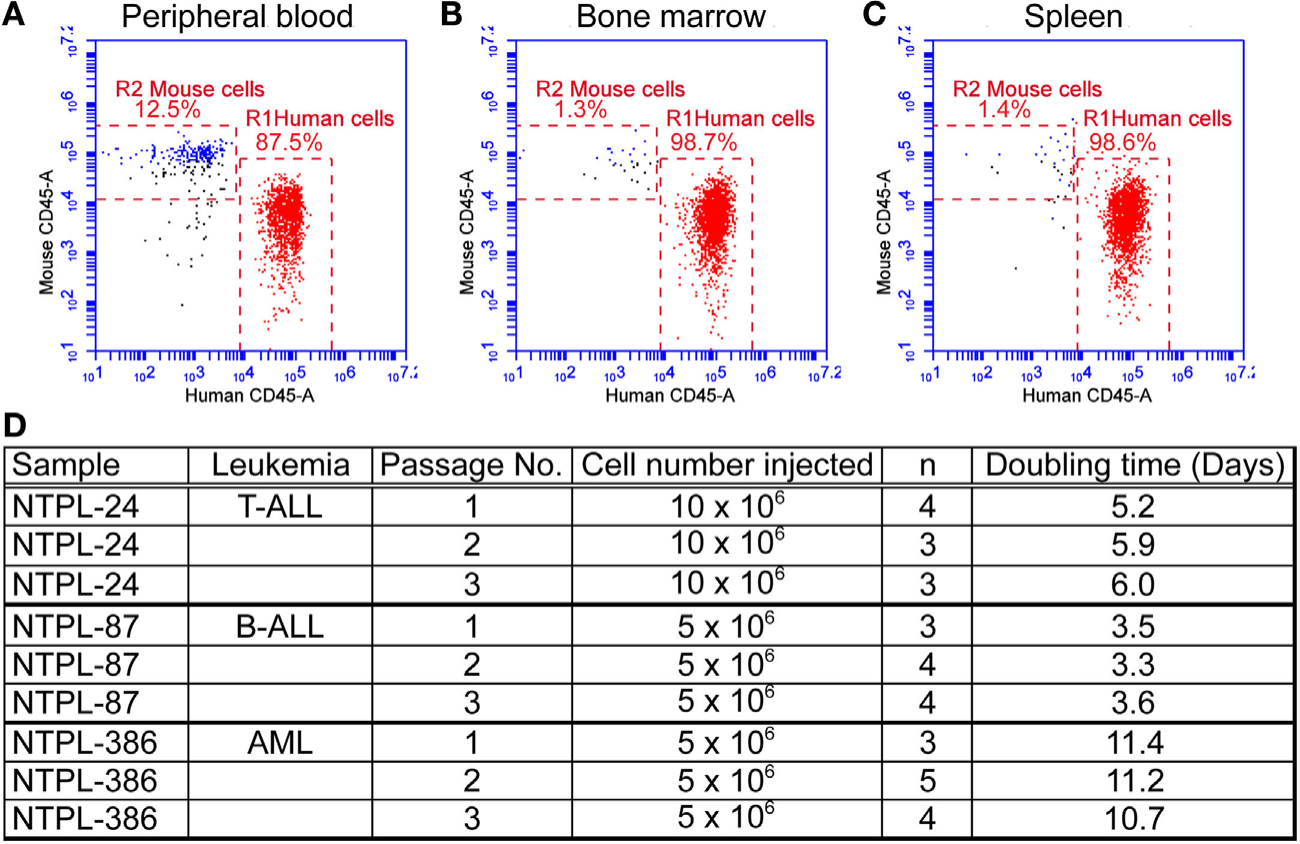
**Primary leukemia samples can be serial passaged in NSG-B2m mice**. NSG-B2m mice were injected with NTPL-24 cells. When the percentage of CD45+ cells in mouse peripheral blood reached above 85%, mice were euthanized. Representative flow cytometry plots showing the percentage of human leukemic cells in mouse peripheral blood **(A)**, bone marrow **(B)**, and spleen **(C)** in these mice. **(D)** Table showing the growth characteristics of leukemia samples over multiple passages.

The mice were euthanized when they exhibited morbidity symptoms. At this point, the percentage of human cells in mouse peripheral blood ranged from 60 to 85% depending on the leukemia sample injected. A representative flow cytometry plot with 87.5% leukemic cells in blood from a mouse injected with NTPL-24 cells is shown (Figure 2A). The percentage of engraftment in bone marrow and spleen approached 100% in the case of majority of leukemia samples (Figures 2B,C show representative plots from NTPL-24 xenografts), indicating near complete replacement of the murine hematopoietic system with leukemic cells. Leukemic cells harvested from the spleens of euthanized mice were enriched by Ficoll density gradient centrifugation and utilized for injection of new cohorts of mice for secondary and tertiary passages. The engraftment characteristics were similar across the three passages of NTPL-24 cells (Figure 2D). Doubling times for other primary samples were also consistent over serial passages in NSG-B2m mice. Data for three representative samples are provided (Figure 2D).

### NSG-B2m Model System Is Suitable for Drug Testing

**Figure 3:**
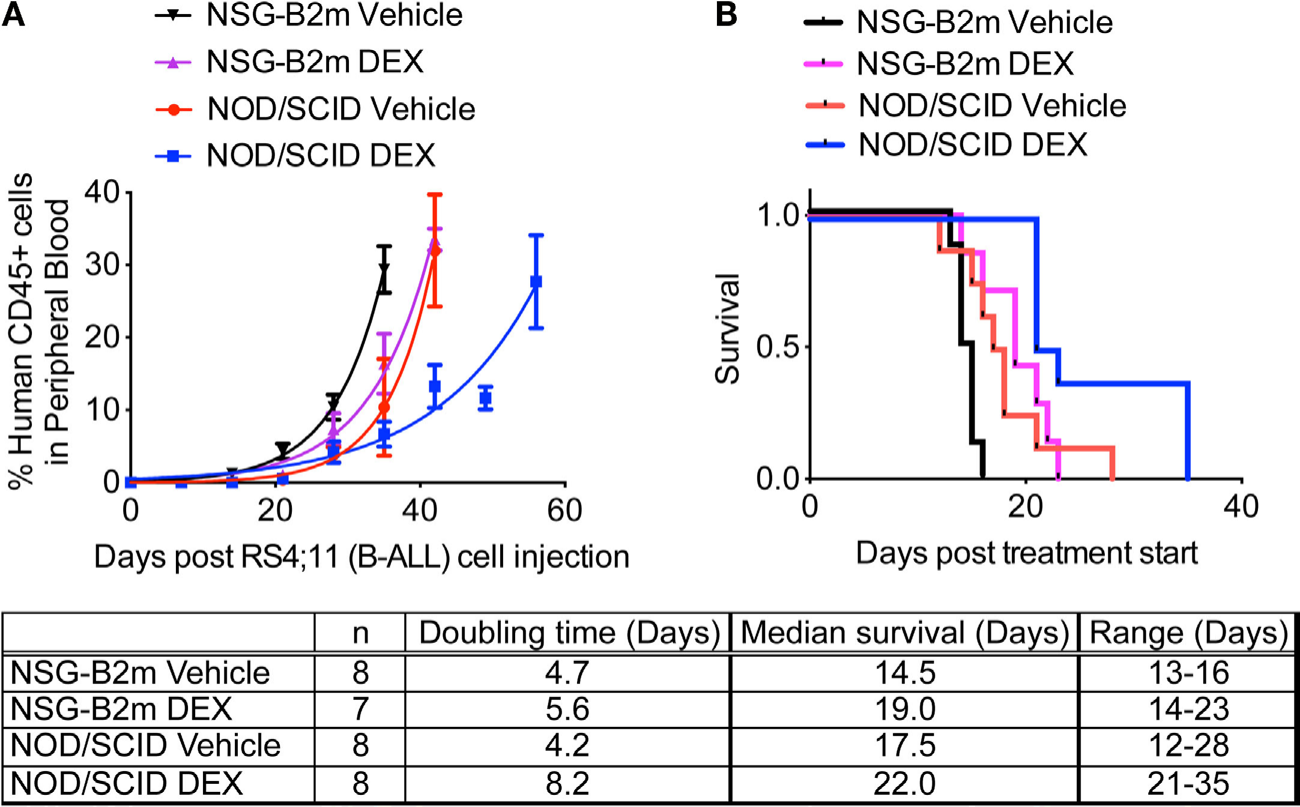
**NSG-B2m model system is suitable for drug testing**. NOD/SCID or NSG-B2m mice were injected with RS4;11 cells. When the median percentage of CD45+ cells in mouse peripheral blood reached above 1%, mice were randomized into two groups and treated with either vehicle or 15 mg/kg dexamethasone (M-F). **(A)** Growth curves showing the rise in percentage of human CD45+ cells. **(B)** Kaplan–Meier survival plots showing the survival of NSG-B2m or NOD/SCID mice following transplantation with RS4;11.

RS4;11 cells are sensitive to dexamethasone *in vitro* and *in vivo* (24, 25). RS4;11 cells (3 × 10^6^) were transplanted in NOD/SCID and NSG-B2m mice, as described above. Once the median percentage of CD45+ human cells in peripheral blood reached 1%, mice were dosed with dexamethasone (15 mg/kg M-F), as described previously (6). Because of their differential growth lag until human cells were detected in mouse blood as described above, treatment was started at 4 weeks for NOD/SCID mice and at 3 weeks for NSG-B2m mice. Dexamethasone treatment significantly increased the doubling time of RS4;11 cells (Figure 3A, *p* < 0.0001 for each model) and conferred a 4.5-day advantage in median survival in both NOD/SCID and NSG-B2m mice (Figure 3B, *p* < 0.005 for NSG-B2m and *p* < 0.01 for NOD/SCID). However, the survival range for dexamethasone-treated NOD/SCID mice was greater than the range of the treated NSG-B2m mice. Further studies are needed to determine if the survival benefit conferred by dexamethasone treatment is comparable between the two models. Nevertheless, these data indicated that NSG-B2m model is useful for testing the efficacy of therapeutics.

### Evaluation of Engraftment Rate of ALL and AML Cell Lines and Patient Samples in NSG-B2m Mice

**Figure 4:**
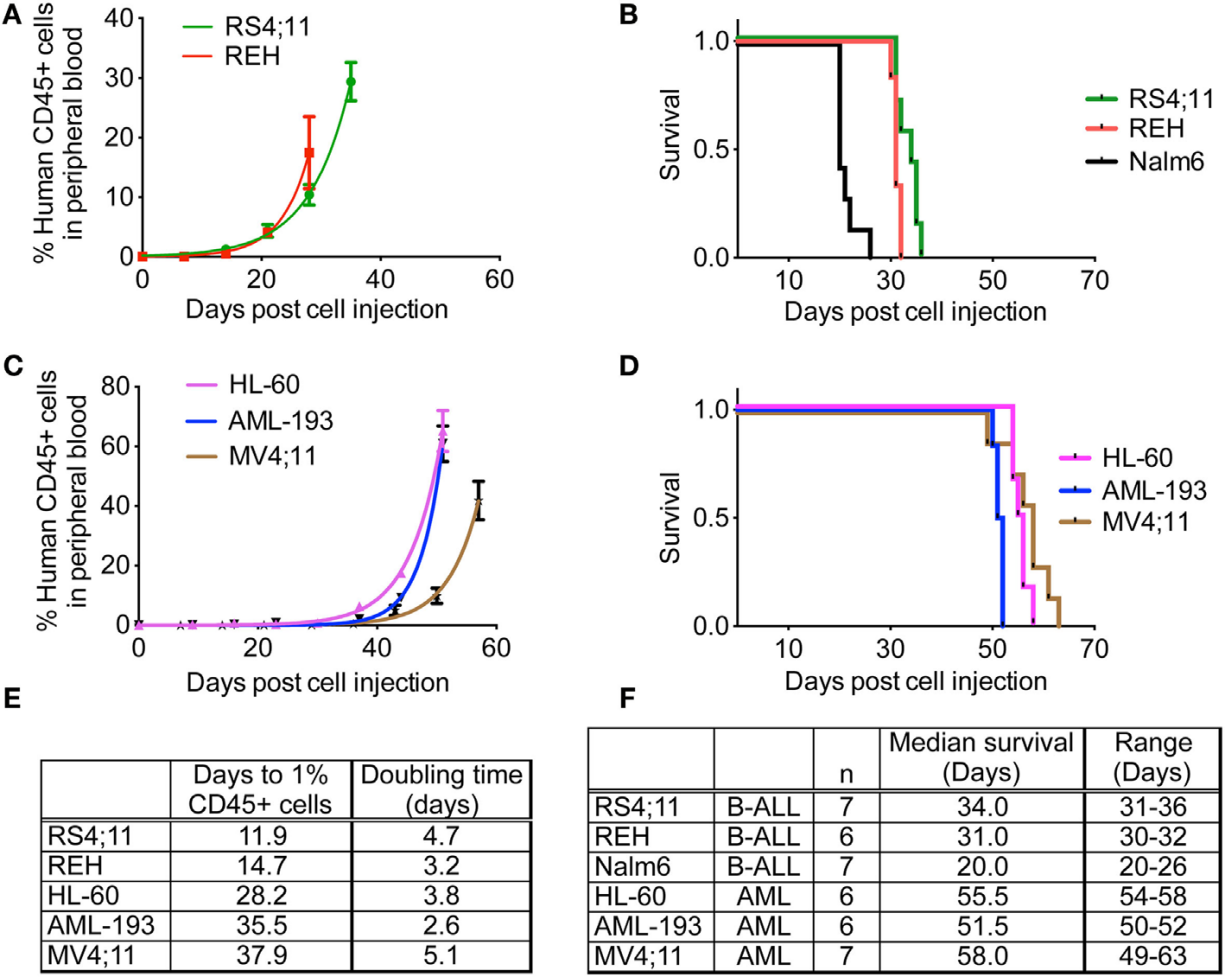
**Engraftment characteristics of ALL and AML cell lines in non-irradiated NSG-B2m mice**. NSG-B2m mice were injected with ALL cell lines (REH, RS4;11, Nalm6) or AML cell lines (HL-60, AML-193, MV4;11). Growth curves show the rise in percentage of human CD45+ cells in peripheral blood in ALL **(A)** and AML **(C)** cell lines. Kaplan–Meier survival plots showing the survival of NSG-B2m mice following transplantation with ALL **(B)** or AML **(D)** cell lines. **(E)** Table summarizing the growth and survival curves in **(A,C)**. **(F)** Table recaptures the growth and survival curves in **(B,D)**.

After establishing NSG-B2m as a better model compared to NOD/SCID mice using a cell line and a patient sample, we subsequently used NSG-B2m mice to determine the engraftment efficiency and kinetics of a variety of cell lines and primary samples in an effort to generate xenograft models for preclinical evaluation of novel drugs. 5 × 10^6^ cells were injected for each sample. The pediatric ALL cell line REH engrafted at a 100% efficiency, with engraftment rate (*p* > 0.01) and median survival (*p* > 0.01) similar to RS4;11 (Figures 4A,B). Nalm6 cells engrafted much faster than REH and RS4;11 and showed a significantly shorter median survival of 21 days (Figure 4F, *p* < 0.0001). Consistent with the reported difficulties in engraftment of AML cells compared to ALL cells, we observed longer lag phases for the three AML cell lines HL-60, AML-193, and MV4;11 tested compared to ALL cell lines (Figure 4E, *p* < 0.0001). HL-60 engrafted most rapidly among the three AML cell lines (Figure 4C, *p* < 0.0001). However, the median survival of HL-60 and MV4;11 cells was similar (Figure 4D, *p* = 0.1299), while the median survival of AML-193 was significantly different than the other two AML cell lines (Figure 4D, *p* < 0.0001).

**Figure 5:**
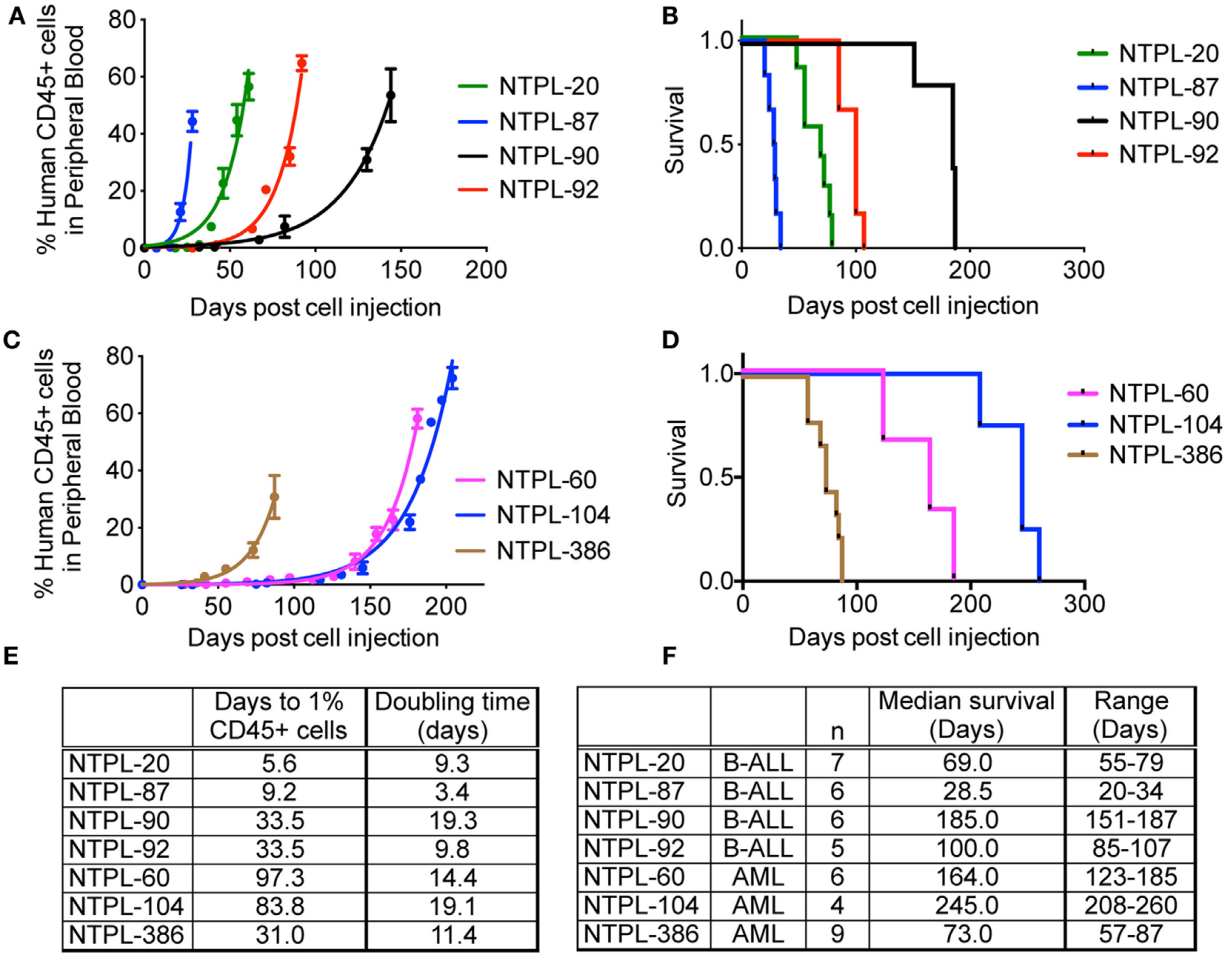
**Engraftment characteristics of primary ALL and AML samples in non-irradiated NSG-B2m mice**. NSG-B2m mice were injected with primary ALL samples (NTPL-20, NTPL-87, NTPL-90, NTPL-92) or primary AML samples (NTPL-60, NTPL-104, NTPL-386). Growth curves show the rise in percentage of human CD45+ cells in peripheral blood in primary ALL **(A)** and AML **(C)** samples. Kaplan–Meier survival plots showing the survival of NSG-B2m mice following transplantation with primary ALL **(B)** or AML **(D)** samples. **(E)** Table summarizing the growth and survival curves in **(A,C)**. **(F)** Table recaptures the growth and survival curves in **(B,D)**.

Encouraged by the 100% engraftment efficiency observed in ALL and AML cell lines, we tested the efficiency of engraftment of primary leukemia cells from the bone marrow aspirates obtained from pediatric patients under IRB approved protocol. Data for engraftment of four ALL and three AML patient samples are shown in Figure 5. Similar to the cell lines, we observed a delay in the engraftment of 2/3 AML primary samples compared to ALL primary samples (Figures 5A,C, *p* < 0.0001). Surprisingly, NTPL-386, a primary AML sample, engrafted rapidly with rates similar to primary ALL samples, NTPL-90 and NTPL-92 (Figures 5B,D). In agreement with the faster engraftment rate, NTPL-386 transplanted mice also died sooner, with a median survival of only 73 days (Figures 5D,F).

### Determination of Immunophenotype of Engrafted Samples

**Figure 6:**
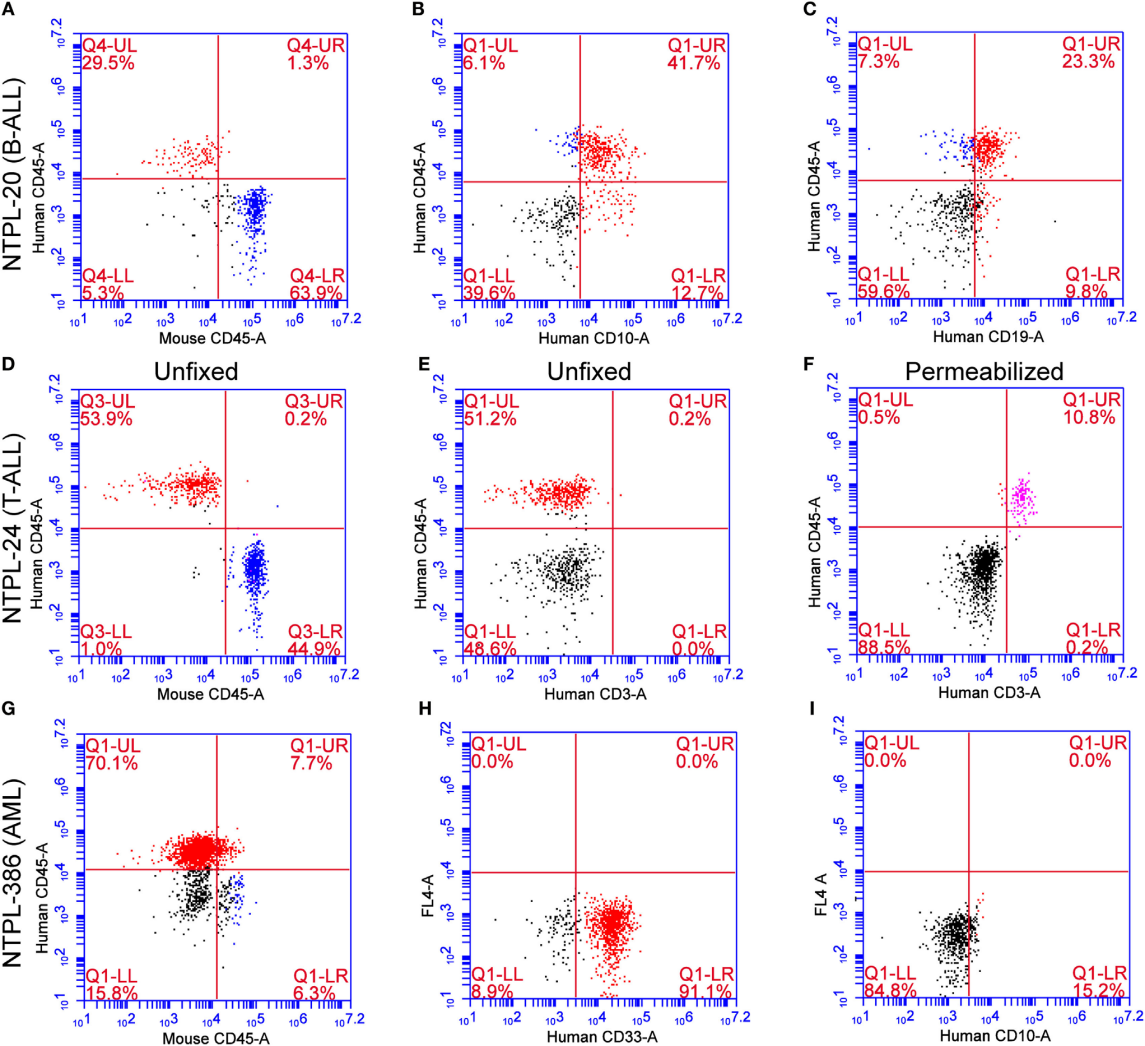
**Immunophenotype confirmation of engrafted primary ALL and AML samples**. Representative flow cytometry plots from peripheral blood of NSG-B2m mice injected with primary leukemia samples (NTPL-20, NTPL-24, NTPL-386) stained with the indicated fluorophore-conjugated antibodies (see axis labels). NTPL-20 showed immunoreactivity for CD45 **(A)**, CD10 **(B)**, and CD19 **(C)**, as expected for a precursor B-ALL sample. NTPL-24 (T-ALL) without or with fixation and permeabilization showed the absence of surface CD3 **(E)** but positivity for intracellular CD3 **(F)**, respectively. AML sample NTPL-386 was positive for CD45 **(G)** and CD33 **(H)** but lacked CD10 immunoreactivity **(I)**. **(A–G)** were double stained for the indicated antibodies, where as **(H,I)** were single stained with a single antibody indicated on the *X*-axis. FL-4 on the *Y*-axis indicates that fluorescent laser 4 was used to generate the scatter plots in **(H,I)**.

To rule out the possibility that the CD45+ human cells arose from engraftment of non-malignant cells in primary samples, we performed immunophenotype determination and characterization of each primary sample. Flow cytometry data on representative samples isolated from the peripheral blood of transplanted mice are presented here (Figure 6). For each sample, we readily distinguished between mouse and human CD45+ cells using species-specific antibodies with minimal cross-reactivity (Figures 6A,D,G). Antigens CD10 and CD19 are co-expressed by the earliest B-cell progenitors and are detected on majority of B-precursor ALL cells (26). NTPL-20 B-precursor ALL xenograft cells showed CD10 and CD19 positivity, which matched with the diagnostic flow cytometry data on patient sample (Figures 6B,C). CD3 T-cell receptor antigen is detected in the cytoplasm of prothymocytes and precursor T-cells and is found on the cell surface of mature T-cells. NTPL-24 precursor T-ALL sample was negative for surface CD3 (Figure 6E) but exhibited cytoplasmic CD3 positivity following fixation and permeabilization of blood (Figure 6F). CD33 antigen is expressed by 85–90% of AML cases and is even used as a target for antibody-based therapies (27). NTPL-386 AML sample was positive for surface CD33 (Figure 6H) but negative for surface CD10 (Figure 6I) in agreement with the flow cytometry profile of patient sample collected at diagnosis. Taken together, these data indicate that the primary leukemic cells engrafted in NSG-B2m mice are neoplastic and faithfully mimic the immunophenotype of the diagnostic patient sample.

## Discussion

The identification of the SCID mutation in the *Prkdc* gene in mice that severely impaired lymphopoiesis (28) prompted the use of SCID mice to engraft B-precursor ALL cells (29). Later studies showed that mice with SCID mutation on NOD background (NOD/SCID) had reduced SCID leakiness and defects in innate and adaptive immunologic functions (16). NOD/SCID mice showed improved engraftment efficiency of ALL and AML cells (30, 31). These models were improved (i) by utilizing transgenic mice deficient in key genes of the adaptive and innate immunity (18, 19, 21, 32), (ii) by host preconditioning with sublethal irradiation or treatment with antibodies against natural killer cells (33), (iii) by treating mice with human cytokines or growth factors (34), or (iv) by generating transgenic mice expressing human growth factors (35). Thus, these improved models created mice that are practically incapable of rejecting human xenografts. We are the first group to utilize non-irradiated NSG-B2m mice for engraftment of pediatric acute leukemia cells.

Since our objective was to generate cell line-derived or patient-derived leukemia xenograft models for preclinical drug testing, we chose to compare the engraftment efficiency between NSG-B2m mice and NOD/SCID mice that are the preferred model for preclinical testing of drugs for pediatric leukemia (15). Several groups have reported 70–90% engraftment efficiency for primary pediatric leukemia samples in non-irradiated NSG mice (19, 36–38), whereas we achieved 100% engraftment in NSG-B2m. That NSG mice were the most efficient disease model compared to NOD/SCID or NS-B2m was demonstrated by direct comparison of the engraftment efficiency (19). Engraftment of HL-60 cells in NOD/SCID mice was 86% (19). In our hands, NSG-B2m mice showed 100% engraftment efficiency for HL-60 cells, indicating an advantage of the non-irradiated NSG-B2m mouse model over NSG model for studying acute leukemia. Improved engraftment efficiency for xenografts was observed in NSGS mice, which are NSG mice with constitutive expression of three human cytokines – SCF, GM-CSF, and IL-3 (35). Future studies with direct comparison between NSG, NSGS, and NSG-B2m mice are required to determine whether NSG-B2m mice engraft better than NSG or NSGS mice.

The severity of the disease was reflected in the engraftment potential of the mice. High-risk and relapsed leukemias tend to engraft very well in mice; however, good prognosis ALL typically took longer or did not engraft at all (30). Primary ALL cells isolated from patients with early relapse showed rapid engraftment and development of leukemia (39) associated with reduced apoptosis signaling (40). The higher engraftment rate of poor-outcome ALL samples could be because of the very high frequency of leukemia stem cells in these samples (20). Similarly, AML engraftment in immunodeficient mice reflected the patient outcome i.e., AML samples that engrafted had a poorer prognosis (41, 42). Using NSG-B2m mice, we were able to engraft primary leukemia samples with variable risk. We also observed that high-risk samples, such as NTPL-20 and NTPL-87, engrafted faster than samples like NTPL-90 with intermediate risk (Figures 5A,E).

The growth rate (or doubling time) in mice has been shown to be dependent on the specific leukemia sample (5). In our study, the doubling time of RS4;11 cells in NOD/SCID and NSG-B2m mice was similar (Figure 1E). NTPL-24 cells also grew at a similar growth rate in NOD/SCID or NSG-B2m mice after the initial lag phase, which was variable. On the other hand, two primary leukemia samples with similar lag phase showed varying growth rate in NSG-B2m mice (Figures 5A,E, compare NTPL-90 and NTPL-92), confirming that the growth rate is an inherent characteristic of the leukemia sample and does not depend on the model used.

Furthermore, the ability to amplify primary patient samples by serial passage is crucial to generate xenograft models for preclinical drug testing. We have utilized this model for testing the efficacy of epigenetic drug combination (43), and for deciphering the role of annexin II/p11 in homing and engraftment to the bone marrow (32). In conclusion, non-irradiated NSG-B2m mouse model faithfully replicates the disease and is an efficient model system for engraftment of primary acute leukemia samples, for studying the disease biology, and for the preclinical drug testing.

## Author Contributions

AG, EK, and SB conceived and designed the experiments. AG, PD, AB, and SB performed the experiments. AG, PD, AB, RM, and SB analyzed the data. AG, EK, RM, DC, and SB contributed reagents and materials. AG and SB wrote the paper.

## Conflict of Interest Statement

The authors declare that the research was conducted in the absence of any commercial or financial relationships that could be construed as a potential conflict of interest.
